# The detection of honey bee (*Apis mellifera*)*-*associated viruses in ants

**DOI:** 10.1038/s41598-020-59712-x

**Published:** 2020-02-19

**Authors:** Alexandria N. Payne, Tonya F. Shepherd, Juliana Rangel

**Affiliations:** Department of Entomology, Texas A&M University, 2475 TAMU, College Station, TX 77843-2475 USA

**Keywords:** Microbial ecology, Behavioural ecology

## Abstract

Interspecies virus transmission involving economically important pollinators, including honey bees (*Apis mellifera*), has recently sparked research interests regarding pollinator health. Given that ants are common pests within apiaries in the southern U.S., the goals of this study were to (1) survey ants found within or near managed honey bee colonies, (2) document what interactions are occurring between ant pests and managed honey bees, and 3) determine if any of six commonly occurring honey bee-associated viruses were present in ants collected from within or far from apiaries. Ants belonging to 14 genera were observed interacting with managed honey bee colonies in multiple ways, most commonly by robbing sugar resources from within hives. We detected at least one virus in 89% of the ant samples collected from apiary sites (n = 57) and in 15% of ant samples collected at non-apiary sites (n = 20). We found that none of these ant samples tested positive for the replication of Deformed wing virus, Black queen cell virus, or Israeli acute paralysis virus, however. Future studies looking at possible virus transmission between ants and bees could determine whether ants can be considered mechanical vectors of honey bee-associated viruses, making them a potential threat to pollinator health.

## Introduction

Positive-sense, single-stranded RNA viruses make up the largest group of honey bee (*Apis mellifera*) infecting pathogens worldwide^1^. Six of the most commonly occurring honey bee-associated viruses include Deformed wing virus (DWV), Black queen cell virus (BQCV), Israeli acute paralysis virus (IAPV), Acute bee paralysis virus (ABPV), Kashmir bee virus (KBV), and Sacbrood virus (SBV)^2^. Although the above listed are commonly referred to as honey bee viruses, previous research has detected these viruses in a number of other arthropods including the ectoparasitic mite *Varroa destructor*^3,4^, other insect pollinators such as hoverflies, bumblebees, and solitary bees^5–19^, and other Hymenopteran insects including some wasps and ants^15,20–28^. Both direct and indirect interactions between honey bees and some of these arthropods (e.g., foraging at the same floral resource, parasitism, and predation) have been proposed as possible routes in which interspecies transmission of honey bee-associated viruses can occur^29–32^.

In the southern United States, ants are ubiquitous within apiaries and are common pests of managed honey bees^33^. However, despite their abundance, few studies have focused on identifying the ants that are common pests of honey bee colonies, or how ant pests interact with managed honey bees, especially in regards to interspecies virus transmission and the impact it might have on honey bee health. The first study to detect the replication of a honey bee-associated virus in an ant was conducted in France, where they found both the viral and replicative genome of Chronic bee paralysis virus (CBPV) in the carpenter ant, *Camponotus vagus*^24^. A later study conducted in North America that screened for honey bee-associated viruses in arthropods found near apiaries (including the carpenter ant, *Camponotus* sp., and the pavement ant, *Tetramorium caespitum*) detected the presence of DWV, BQCV, IAPV, and SBV in *Camponotus* individuals. However, they did not detect any virus replication in the ants sampled^23^. Two studies conducted in New Zealand detected the presence of DWV, BQCV, and KBV, as well as the replicative form of DWV and KBV, in the Argentine ant, *Linepithema humile*^21,22^. In another study, Lake Sinai virus (LSV) and phylogenetically related viruses were detected in three species of harvester ants including *Messor concolor*, *M*. *barbarus*, and *M*. *capitatus*^28^. Moreover, it was recently found that *Myrmica rubra* ants collected in Berlin, Germany can be infected with types A and B of DWV when fed infected honey bee pupae in caged environments^27^.

In most of the above studies, ants were tested for the detection of honey bee-associated viruses without reporting how the ants naturally interacted with managed honey bees. To better understand which ants are pests within apiaries and how these pests interact with and potentially impact managed honey bees, we (1) surveyed and identified ants collected in or near apiaries in southern and central Texas, (2) documented the type of interactions observed between ants and managed honey bees, and (3) screened for the presence of DWV, BQCV, IAPV, ABPV, KBV, and SBV and the replication of DWV, BQCV, and IAPV in ants collected from apiary and non-apiary sites. Our study revealed a number of ant taxa that act as common pests of honey bees within apiaries and explored whether or not these ants may act as hosts of six honey bee-associated viruses, which could have important implications regarding honey bee health.

## Results

### Sample collection

We collected a total of 77 ant samples between 2017 and 2018 throughout Texas. A sample consisted of a group of individuals that belonged to the same taxon and were in the same life stage (i.e. all immatures or all adults) and were collected at the same site, on the same day, and from the same hive or nearby ant colony. Of the 57 samples collected at apiary sites, 26 were collected directly from within or on honey bee hives. The remaining 31 samples were collected within 20 meters of honey bee colonies, including structures or areas where beekeepers stored equipment such as unused hive bodies or frames. We identified a total of 14 ant genera, with the most common taxa being *Solenopsis invicta* (fire ants) and *Crematogaster* sp. (acrobat ants). In addition to ants collected within apiaries, 20 ant samples belonging to six different genera were collected from non-apiary sites to compare the presence of viruses between the two types of locations. A summary of the ant taxa collected from apiary and non-apiary sites is listed in Table 1.

**Table 1:** Summary of ant samples collected from apiary and non-apiary locations throughout central Texas.

	Ant taxa collected	Number of samples	Number (%) of samples that tested positive for a virus					
			DWV	BQCV	IAPV	ABPV	KBV	SBV
Apiary sites (n = 57)	*Aphaenogaster texana* (spine-waisted ant)^a^	1	0	0	0	1 (100%)	0	0
	*Brachymyrmex* sp. (rover ant)^a,b^	2	2 (100%)	1 (50%)	0	0	0	1 (50%)
	*Camponotus* sp. (carpenter ant)^d^	4	3 (75%)	1 (25%)	1 (25%)	1 (25%)	0	0
	*Crematogaster* sp. (acrobat ant)^a^	17	11 (64.7%)	9 (52.9%)	4 (23.5%)	11 (64.7%)	1 (5.9%)	3 (17.6%)
	*Forelius* sp. (cheese ant)^b^	1	1 (100%)	0	0	0	0	0
	*Formica* sp. (field ant)^**c**^	1	0	0	0	0	0	0
	*Linepithema humile* (Argentine ant)^b,e,f^	2	1 (50%)	0	0	1 (50%)	1 (50%)	0
	*Monomorium minimum* (little black ant)^b,c^	0	—	—	—	—	—	—
	*Nylanderia sp*. (crazy ant)^b,e^	1	0	1 (100%)	1 (100%)	1 (100%)	0	0
	*Pheidole sp*. (big headed ant)^c^	4	3 (75%)	2 (50%)	1 (25%)	0	0	0
	*Pogonomyrmex sp.* (harvester ant)	1	0	0	0	1 (100%)	0	0
	*Pseudomyrmex gracilis* (elongate twig ant)	4	3 (75%)	0	0	0	0	0
	*Solenopsis invicta* (fire ant)^b,c,d,f,g^	18	14 (77.8%)	8 (44.4%)	5 (27.8%)	7 (38.9%)	4 (22.2%)	8 (44.4%)
	*Tapinoma sp*. (odorous house ant)	1	0	0	0	0	0	0
	Total number of samples	57	38 (67%)	22 (39%)	12 (21%)	22 (39%)	6 (11%)	12 (21%)
Non-apiary sites (n = 20)	*Brachymyrmex sp*. (rover ant)	1	0	0	0	0	0	0
	*Crematogaster sp*. (acrobat ant)	3	0	0	0	0	0	0
	*Nylanderia sp*. (crazy ant)	1	0	0	0	0	0	0
	*Pheidole sp*. (big headed ant)	3	0	0	0	0	0	0
	*Pseudomyrmex gracilis* (elongate twig ant)	1	0	0	0	0	0	0
	*Solenopsis invicta* (fire ant)	11	3 (27.3%)	0	0	0	2 (18.2%)	0
	Total number of samples	20	3 (15%)	0	0	0	2 (10%)	0

The table includes information on the ant taxa collected, the different interactions observed between ants and managed honey bees within/near hives (denoted as superscript numbers alongside ant common names), and the prevalence of six honey bee-associated viruses in the sampled ants after performing diagnostic analysis using RT-PCR. A number of interactions were observed between ants and honey bees within managed apiaries including: ^a^cohabitation of honey bees and ants (including brood and reproductives) within the same honey bee hive; ^b^robbing of sugar resources (e.g., nectar, honey, and/or beekeeper-supplied sugar syrup) by ants from within the hive; ^c^robbing of pollen; ^d^foraging for honey/sugar from beekeeping equipment and/or supplies; ^e^causing a honey bee colony to abscond due to an overwhelming level of robbing behavior by ants; ^f^scavenging of dead adult bees; and ^g^preying on honey bee brood or removing brood from the colony. Ants without a number indicating an interaction type were collected on or near a honey bee hive but were not observed interacting with the bee colony in any way. Viruses that were screened from collected ant samples included Deformed wing virus (DWV), Black queen cell virus (BQCV), Israeli acute paralysis virus (IAPV), Acute bee paralysis virus (ABPV), Kashmir bee virus (KBV), and Sacbrood virus (SBV). Of the 57 ant samples that were collected from within apiaries and provided viable RNA, 51 (89%) tested positive for at least one virus of interest. For ants collected at non-apiary sites, only 3 of the 20 samples (15%) tested positive for at least one virus of interest. In many instances, a single ant sample tested positive for multiple viruses. The table does not include virus information for Monomorium minimum, as none of those samples provided viable RNA. Samples that tested positive for DWV, BQCV, and IAPV after the diagnostic RT-PCR were then analyzed for the replication of these viruses by strand-specific RT-PCR. Data on the replication of viruses were not included, as they were negative for all tested samples.

### Interactions observed between honey bees and ants

**Figure 1:**
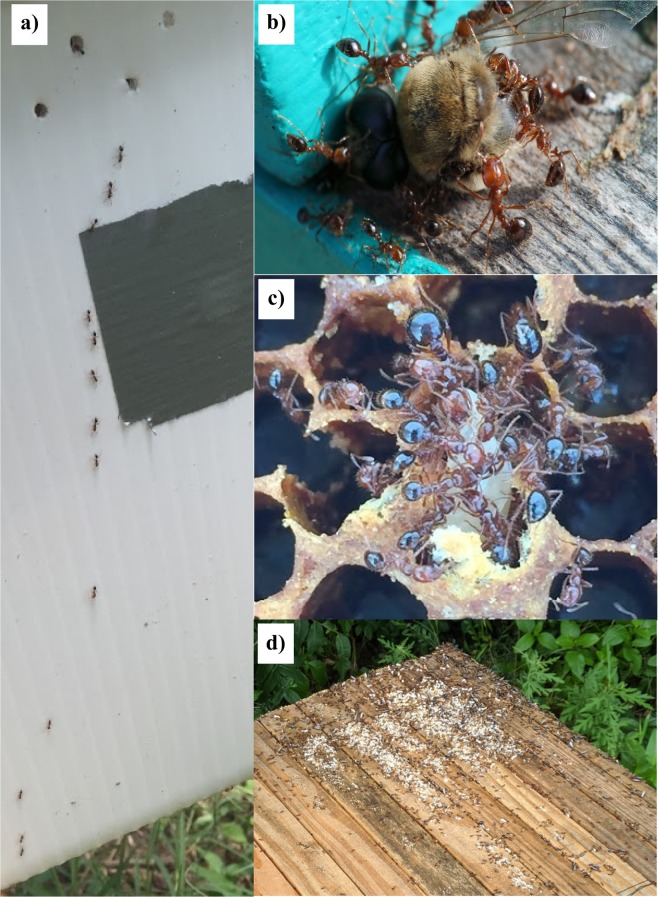
Depiction of some of the different interaction types observed between honey bees and ants within apiaries including (**a**) predation: *Solenopsis invicta* workers removing a bee larva from within a cell in a collapsed hive; (**b**) scavenging: *S. invicta* transporting the head and thorax of a dead adult drone out of a hive’s entrance (photo credit: Pierre Lau); (**c**) co-habitation: a *Crematogaster* sp. colony living within a honey bee top-bar hive (photo credit: Pierre Lau); and (**d**) robbing of hive resources: *Crematogaster* sp. foraging trail leading out of a honey bee nucleus colony.

Ants at apiary sites were observed interacting with managed honey bees in multiple ways including robbing sugar or pollen resources from within the hive, scavenging dead honey bee adults, preying on honey bee brood, and cohabiting with bees within the hive (Fig. 1). The most common interaction observed between ants and honey bees was the robbing of sugar resources including nectar, honey, and/or beekeeper-supplied sugar syrup (Fig. 1a). Ants in the genera *Brachymyrmex*, *Forelius, Linepithema*, *Monomorium*, *Nylanderia* and *Solenopsis* were observed either foraging from beekeeper-supplied feeders or within wax cells where honey bees stored nectar. In some instances, entire bee colonies abandoned their hives (i.e., absconded) due to high rates of robbing, done mostly by ants with large populations such as *Nylanderia fulva* and *Linepithema humile*. Ants in the genera *Formica*, *Monomorium*, *Pheidole*, and *Solenopsis* were also observed robbing and transporting pollen out of hives.

*Camponotus* and *Solenopsis* ants were observed scavenging dead adult bees from within or near hives (Fig. 1b), while *S. invicta* was the only species observed preying on bee brood (Fig. 1c). This behavior was common for colonies that were weak or those that had absconded or collapsed and contained abandoned brood. Small colonies of *Brachymyrmex* ants were found living on top of hives, typically underneath bricks that were placed to keep the hive lids from being blown off by the wind. Only ants in the genus *Crematogaster* were found living within honey bee hives (Fig. 1d). This included whole ant colonies containing eggs, immatures, workers, and reproductives that lived either between the outer and inner covers of a hive or within tunnels that they had created through the wood of hive boxes.

### Diagnostic analysis of honey bee-associated viruses in ants

Of the 14 ant genera associated with honey bees that were collected within apiaries, 13 were screened for the presence of viruses. *Monomorium minimum* ants were excluded from the analysis due to their small body mass and the inadequate number of individuals collected per sample, which resulted in low RNA yield. Of the 57 ant samples collected from within apiaries, 51 (89%) tested positive for at least one virus. In many cases, we detected multiple viruses in a single ant sample. The most prevalent virus in ants collected from apiaries was DWV, with 38 of the 57 samples (66.7%) testing positive (Table 1). The least prevalent virus was KBV, with only six of the 57 samples (10.5%) testing positive. At non-apiary sites, only DWV and KBV were detected in *S. invicta* ants, with three of the 20 samples (15%) testing positive for DWV and two (10%) testing positive for KBV.

### No replication of DWV, BQCV, or IAPV detected in ants

When conducting the initial strand-specific RT-PCR to test for replication of DWV, BQCV, and IAPV, one sample had tested positive for replicating DWV, and four samples tested positive for replicating IAPV. All samples that had tested positive for viral replication belonged to the genus *Crematogaster*, including one that consisted entirely of immature ants. However, when we tested these five samples again and performed a digest step using Exonuclease I and a 10-fold dilution following reverse transcription, we found that all of the previously positive samples tested negative for replication and were considered to originally have yielded false positive results, indicating a lack of viral replication in any of the ant samples collected from either apiary or non-apiary locations.

## Discussion

Of the 500+ described ant species present in the United States, nearly 300 occur in the warm, subtropical climate of Texas, with around 20 species considered non-native pests^34^. Personal communications with commercial and backyard beekeepers (e.g., members of the Texas Beekeepers Association) made us aware of how common ants are within managed apiaries. Depending on the genus, ants have been observed robbing sugar resources and pollen from within hives, preying on bee brood, scavenging dead adult bees, or cohabiting with honey bees within their hives.

The ants most frequently found within apiaries were *S. invicta* and *Crematogaster* sp., both of which are common in Texas. Fire ants were often observed preying on bee brood and deceased adults, especially in hives that had collapsed or were weak and close to collapsing. For instance, it is common practice by beekeepers in Texas to place a frame of honeycomb infested with secondary pests (e.g., the greater and lesser wax moths, *Galleria mellonella* and *Achroia grisella*) or diseased brood onto a *S. invicta* mound for the ants to “clean out” the comb for future use (Gene Ash, pers. comm.). In the case of acrobat ants, entire colonies, including immatures and reproductives, were found inhabiting hives between the inner and outer covers. In two different hives we observed that a colony of *Crematogaster* sp. had created tunnels and was dwelling within the wood of a hive box. This genus is typically arboreal but has been known to live within dead wood on the ground^35^.

Other less commonly encountered ants within apiaries were two invasive species from South America that can reach large population densities in the United States: *Nylanderia fulva* (tawny crazy ants) and *Linepithema humile* (Argentine ants). In two of the 21 apiary sites we sampled from, honey bee colonies absconded due to overwhelming nectar robbing by invading tawny crazy ants or Argentine ants. Previous reports have documented the eventual absconding or collapse of honey bee colonies when they are invaded and overrun by these ants if they reach overwhelmingly large population densities^36^.

The various associations we observed between ants and honey bees are potential routes for interspecies transmission of honey bee-associated viruses between these eusocial insects. A growing body of research has recently focused on the detection of honey bee-associated viruses in other arthropod groups^3–28^. This area of research is particularly important in the context of honey bee pathogen transmission given that viruses can rapidly mutate and adapt to novel hosts, particularly those that are genetically similar or whose biological niche overlaps with that of the original host. This rapid adaptation of viruses could have ecological consequences such as influencing changes in the structure of a community containing susceptible insects^37^.

It should be noted, however, that the identification of a virus in a novel host species is not indicative of a “spillover” event, the process through which a virus is transmitted from a reservoir population into a native or novel host. Instead, at least in the case of honey bee-associated viruses, it is more likely that spillover would have first occurred in susceptible hosts when honey bees were first introduced into the New World by European settlers during the seventeenth century^38^. Any spillover event would have likely occurred in genetically similar species that had overlapping floral resources with honey bees (e.g., bumblebees and solitary bees), such that viruses could have been transmitted through the sharing of nectar or pollen^15,39^. The growing number of novel host species that have recently been found to foster honey bee-associated viruses speaks more about our advances in detecting these viruses then it does about the occurrence of a recent spillover event.

We discovered that 51 of 57 ant samples collected at apiary sites (89%) and three of 20 samples collected at non-apiary sites (15%) tested positive for the presence of one or more virus. Because the three samples collected from non-apiary sites were all *S. invicta*, we hypothesize that these omnivorous ants picked up the viruses by scavenging bee foragers that had died away from their hive. However, none of these samples tested positive for replication of DWV, BQCV, or IAPV. Overall, previous studies have tested only a relatively few number of ant species for the replication of DWV, BQCV, or IAPV^21–23,27^. Argentine ants are one of the few ant species that have been tested for a honey bee-associated virus (DWV) in multiple areas worldwide including Argentina, New Zealand, Australia, and now the United States. Yet, only ants collected in New Zealand have been shown to have the replicative form of DWV^21,22^. This indicates that replication of honey bee-associated viruses in this species may be due to genetic variation of the virus, the ant, or both, as a result of geographical location. Argentine ants are not as common in Texas as in other areas of the United States, so it would be interesting to sample this species in a broader geographical range with an increased sample size in order to better answer this question.

Beyond being a pest for beekeeping operations, ants may be impacting bee health in more ways than previously thought. Despite lack of viral replication, ants that feed on infected honey bee brood or adults, or on infected sugar and pollen resources, may still act as mechanical vectors of honey bee-associated viruses. It is speculated that virus-containing ants disseminate viruses to honey bees by invading hives and transmitting the viruses to nectar or honey cells while robbing, which can then enter bees that subsequently feed from these cells. This is especially likely of ants that are common pests within hives, such as *S. invicta* and *Crematogaster* sp. For instance, a previous study showed that ants can acquire honey bee-associated viruses through foodborne transmission (i.e., the ingestion of infected honey bee pupae)^27^. However, further research looking at the possible transmission mechanisms of these viruses from ants to honey bees is needed to determine whether or not ants play a role in transmitting viruses to honey bees, which would contribute to the declining health of this important pollinator.

## Methods

### Sample collection

We began our study with a survey to identify the ants that act as pests of managed honey bee colonies. We collected a total of 57 ant samples from January 2017 to September 2018 from 21 apiaries across Texas where beekeepers had reported having issues with ants living within or around their hives. A sample consisted of individuals that belonged to the same taxon and were collected at the same site, on the same day, and from the same honey bee hive or nearby ant colony. The number of individuals per sample ranged from one ant (e.g., species with solitary foraging habits), to a few hundred individuals (e.g., ant nests with high population densities). If a sample contained individuals at different life stages (i.e., immatures vs. adults), it was further divided into two distinct samples. Samples were collected with forceps and an aspirator either from within/on honey bee hives or from locations within 20 meters of a managed honey bee colony.

To better understand the extent to which honey bee-associated viruses are present in ants, 20 ant samples were collected from nine sites located at least 3.2 km away from any managed honey bee colonies (non-apiary sites). All samples were stored in 15 mL centrifuge tubes on dry ice upon collection in the field to maintain RNA integrity before being stored at −80 °C in the laboratory. Ants were identified using printed keys and specimens from Texas A&M University’s insect collection^40,41^.

### RNA extraction

Each sample, consisting of whole-bodied ants, was homogenized in an Eppendorf tube using a pestle. Up to 20 mg of the homogenate was then used for total RNA extraction (Aurum Total RNA Mini Kit, Bio-Rad Laboratories, Hercules, CA), which included a DNase digestion step. The extracted RNA was eluted into a 40 µL solution and tested for its concentration and purity on a NanoPhotometer NP80 (Implen, Munich, Germany) before being stored at −80 °C.

### Diagnostic analysis for honey bee-associated viruses

The extracted total RNA underwent diagnostic analyses for common honey bee-associated viruses including DWV, BQCV, IAPV, ABPV, KBV, and SBV. To accomplish this, 250 ng of total RNA was first reverse transcribed with random primers (250 nM final concentration) in a 20 µL reaction (iScriptSelect cDNA Synthesis Kit, Bio Rad Laboratories, Hercules, CA). PCR amplification was performed with *Taq* DNA Polymerase (New England Biolabs, Ipswitch, MA). Virus-specific primers commonly used to screen honey bees for these viruses (Table 2), as well as cloned PCR products corresponding to each primer set that were used as positive controls, were acquired from the USDA-ARS Bee Research Laboratory in Beltsville, MD. The acquired PCR products that were used as positive controls can be visualized in the Supplemental Fig. S1. PCR cycling conditions included an initial denaturation step at 94 °C for 2 min, followed by 30 cycles of 94 °C for 30 s, 55 °C for 30 s and 72 °C for 1 min, without a final extension step. The resulting PCR products were visualized on a 3% agarose gel using gel electrophoresis, stained with ethidium bromide, and photographed under UV light. A subset of samples that tested positive for a virus can be visualized in Supplemental Fig. S2. Samples that tested positive for each virus were confirmed via Sanger sequencing.

**Table 2:** List of primers used throughout this study.

Primer #	Primer name	Sequence (5′- 3′)	Amplicon size (bp)	Reference/Source
1	DWV.F.	GAGATTGAAGCGCATGAACA	130	vanEngelsdorp *et al*.^47^
2	DWV.R.	TGAATTCAGTGTCGCCCATA		
3	BQCV.F	TTTAGAGCGAATTCGGAAACA	140	
4	BQCV.R.	GGCGTACCGATAAAGATGGA		
5	IAPV.F.	GCGGAGAATATAAGGCTCAG	587	
6	IAPV.R.	CTTGCAAGATAAGAAAGGGGG		
7	ABPV.F.	ACCGACAAAGGGTATGATGC	124	
8	ABPV.R.	CTTGAGTTTGCGGTGTTCCT		
9	KBV.F.	TGAACGTCGACCTATTGAAAAA	127	
10	KBV.R.	TCGATTTTCCATCAAATGAGC		
11	SBV.F.	GGGTCGAGTGGTACTGGAAA	105	
12	SBV.R.	ACACAACACTCGTGGGTGAC		
13	tag only	agcctgcgcaccgtgg	not applicable	Yue *et al*.^42^
14	tag-DWV F15	agcctgcgcaccgtggTCCATCAGGTTCTCCAATAACGG	451	Yue *et al*.^42^
15	DWV B23	CCACCCAAATGCTAACTCTAACGC		Genersch^45^
16	tag-BQCVsense	agcctgcgcaccgtggTCAGGTCGGAATAATCTCGA	419	Peng *et al*.^8^
17	BQCV-antisense	GCAACAAGAAGAAACGTAAACCAC		
18	tag-IAPVsense	agcctgcgcaccgtggGCGGAGAATATAAGGCTCAG	587	Di Prisco *et al*.^3^
19	IAPV-antisense	CTTGCAAGATAAGAAAGGGGG		

Primer sets 1–12 were used in the diagnostic RT-PCR reactions for Deformed wing virus (DWV; primers 1 and 2), Black queen cell virus (BQCV; primers 3 and 4), Israeli acute paralysis virus (IAPV; primers 5 and 6), Acute bee paralysis virus (ABPV; primers 7 and 8), Kashmir bee virus (KBV; primers 9 and 10), and Sacbrood virus (SBV; primers 11 and 12). Primers 13–19 were used for the detection of the negative sense strand indicative of viral replication using strand-specific RT-PCR. Reverse transcription targeting the negative-sense strand was conducted with primer numbers 14 for DWV, 16 for BQCV, and 18 for IAPV. The tag only primer (13) was the forward primer of all three viruses for PCR reactions, and primers 15, 17, and 19 were the reverse primers for DWV, BQCV, and IAPV respectively.

### Detection of DWV, BQCV, and IAPV replication in ants

The three viruses that were initially screened for and detected in ant samples (DWV, BQCV, and IAPV) were further tested for replication within ants using tagged primers in a modified two-step RT-PCR^42,43^. The use of strand-specific RT-PCR (ssRT-PCR) to detect the replicated intermediate of viruses is described by de Miranda *et al*., 2013^44^. Briefly, 250 ng of total RNA was reverse transcribed with gene-specific primers (250 nM final concentration) in a 20 µL reaction (iScriptSelect cDNA Synthesis Kit, Bio Rad Laboratories, Hercules, CA). To target the negative-sense strand of the virus, only a forward primer complementary to the negative strand specific for either DWV^45^, BQCV^8^, or IAPV^3^ was used in the reaction. Each forward primer contained a tag attached to its 5′ end^42^ to increase the specificity of the primers and thus decrease the possibility of detecting false positives^44^. The PCR reactions were subsequently carried out with a primer pair at a final primer concentration of 10 µM each. These two primers consisted solely of the tag sequence and a virus-specific reverse primer using the following PCR cycle conditions: an initial denaturation step at 94 °C for 2 min, followed by 35 cycles of 94 °C for 30 s, 55 °C for 30 s and 72 °C for 1 min, with a final extension step of 72 °C for 10 min. PCR products were then visualized using gel electrophoresis on a 3% agarose gel. Primers used for the detection of viral replication are listed in Table 2. This method of ssRT-PCR can result in the attainment of false positives due to false-, self-, and mis-priming events that most likely occurs when residual primers from cDNA synthesis are carried over into the PCR reaction. To avoid these potential problems, samples that tested positive for replication underwent a second analysis to confirm the absence of false positives. Reverse transcription was carried out as previously described with additional controls including a template-free control, a RT-free control, and a primer-free control for each tested sample. The resulting tagged cDNA was then treated with an Exonuclease-I (New England Biolabs, Ipswitch, MA) digestion step, which removes excess primers and has been shown to greatly reduce non-specific priming, and then diluted 10-fold prior to performing PCR^44,46^. PCR products were then visualized through gel electrophoresis using a 3% agarose gel.

## Supplementary information

Supporting Information.
