# Supplementary material for: The detection of honey bee (Apis mellifera)-associated viruses in ants
Source: Sci Rep. 2020 Feb 19;10:2923. doi: 10.1038/s41598-020-59712-x (PMC7031503; doi:10.1038/s41598-020-59712-x)
Supplement: Supplementary file 1 — Supporting Information. [file 41598_2020_59712_MOESM1_ESM.docx]

The detection of honey bee (*Apis mellifera*)-associated viruses in ants

Alexandria N. Payne^1^, Tonya F. Shepherd^1^, and Juliana Rangel^1*^

^1^Department of Entomology, Texas A&M University, 2475 TAMU, College Station, TX 77843-2475, USA

^*^To whom correspondence should be addressed: J. Rangel. Department of Entomology, Texas A&M University, 2475 TAMU, College Station, TX 77843, USA. Phone number: 979-845-1074. Fax: 979-845-6305. E-mail: jrangel@tamu.edu


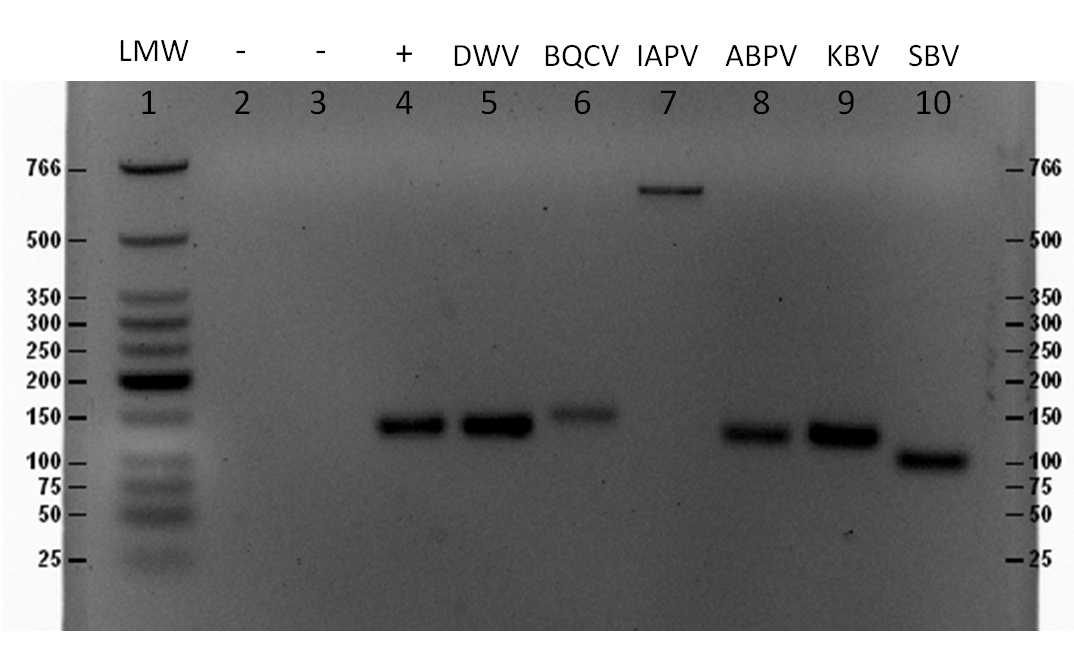


**Supplemental Figure S1:** Cloned PCR products were provided by the USDA-ARS Bee Research Laboratory in Beltsville, MD to serve as positive controls for our diagnostic analyses. Each control correlated to one of our six viruses of interest including Deformed wing virus (DWV; Lane 5), Black queen cell virus (BQCV; Lane 6), Israeli acute paralysis virus (IAPV; Lane 7), Acute bee paralysis virus (ABPV; Lane 8), Kashmir bee virus (KBV; Lane 9), and Sacbrood virus (SBV; Lane 10). A template-free reaction (Lane 2) and a RT-free reaction (Lane 3) served as negative controls when conducting all diagnostic RT-PCRs. Lane 4 consisted of a symptomatic honeybee displaying the crumpled-wing phenotype of DWV and served as another positive control for this virus. All PCR products were run against a low molecular weight ladder (LMW; Lane 1) (New England Biolabs® Inc.). The amplicon sizes of each control were the following: DWV (130 bp), BQCV (140 bp), IAPV (587 bp), ABPV (124 bp), KBV (127 bp), and SBV (105 bp).


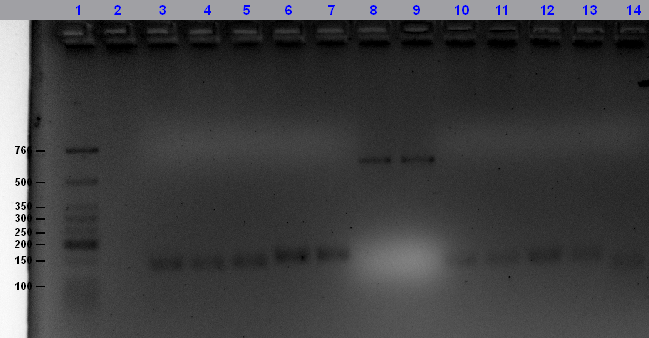


1

2

3

4

5

6

7

8

9

10

11

12

13

14

LMW

-

DWV

BQCV

IAPV

ABPV

KBV

SBV

+

**Supplemental Figure S2:** A subset of samples that tested positive for a virus. This included Deformed wing virus (DWV) in *Brachymyrmex* sp. (Lane 4) and in immature stages of *Crematogaster* sp. (Lane 5), Black queen cell virus (BQCV) in *Solenopsis invicta* (Lane 6) and in adult *Crematogaster* sp. (Lane 7), Israeli acute paralysis virus (IAPV) in *S. invicta* (Lane 8) and in *Pheidole* sp. (Lane 9), Acute bee paralysis virus (ABPV) in two samples of *Crematogaster* sp. (Lanes 10 and 11), Kashmir bee virus (KBV) in two samples of *S. invicta* (Lanes 12 and 13), and Sacbrood virus (SBV) in a sample of *S. invicta* (Lane 14). A template-free reaction served as a negative control (Lane 2), and an adult honey bee with obvious symptomatic infection of DWV served as a positive control (Lane 3). All PCR products were run against a Low Molecular Weight ladder (LMW; Lane 1). The amplicon sizes of each product were the following: DWV (130 bp), BQCV (140 bp), IAPV (587 bp), ABPV (124 bp), KBV (127 bp), and SBV (105 bp). All PCR products were run against a Low Molecular Weight ladder (LMW; Lane 1) (New England Biolabs® Inc.).
